# The Hidden Cost of Immunity: Myocarditis Presenting as Complete Heart Block in an Older Adult Following COVID‐19 Booster

**DOI:** 10.1155/cric/4549069

**Published:** 2026-09-08

**Authors:** Dhan Bahadur Shrestha, Ruvarashe Mupedziswa, Daniel H. Katz, Mustafain Meghani

**Affiliations:** ^1^ Division of Cardiology, Department of Internal Medicine, Bassett Medical Center, Cooperstown, New York, USA, bassett.org; ^2^ Department of Internal Medicine, Bassett Medical Center, Cooperstown, New York, USA, bassett.org

**Keywords:** high-grade atrioventricular block, late gadolinium enhancement, vaccine-induced myocarditis

## Abstract

**Background:**

Myocarditis has been rarely reported following COVID‐19 vaccination, most commonly in younger males. Although a temporal association has been observed, establishing causality in individual cases remains challenging. Myocardial inflammation may involve the cardiac conduction system, rarely resulting in high‐grade atrioventricular (AV) block. Importantly, these conduction abnormalities may be transient and resolve as the underlying inflammation improves.

**Case Summary:**

We report a rare case of myocarditis following a COVID‐19 vaccination in an elderly woman who experienced complete heart block and worsening exertional dyspnea subsequent to receiving a COVID‐19 booster. Coronary angiography revealed patent coronary arteries, and magnetic resonance imaging confirmed the presence of myocarditis. Her conduction abnormality progressively resolved without the need for permanent pacing, and she was subsequently discharged in stable condition.

**Discussion:**

Myocarditis should be considered as a potentially reversible cause of high‐grade AV block. A thorough clinical history and multimodal imaging are essential for diagnosis, as early recognition and appropriate monitoring may allow recovery of AV conduction and avoid unnecessary permanent pacemaker implantation.

**Take-Home Message**

This case highlights the importance of recognizing acute myocarditis as a potentially reversible cause of complete heart block. Early diagnosis and close monitoring of AV conduction may allow for recovery and avoid unnecessary permanent pacemaker implantation. Further keynotes are as follows:

1. High‐grade AV block can be an atypical but reversible manifestation of COVID‐19 vaccine‐associated myocarditis, even in older adults.
2. Cardiac MRI is a critical diagnostic tool for confirming myocarditis in patients presenting with elevated troponins and ischemic symptoms with normal coronary arteries.
3. A thorough clinical history, including recent vaccination, is essential when evaluating patients with new conduction abnormalities or unexplained cardiac symptoms.
4. Empiric treatment for infectious causes such as Lyme carditis may be reasonable initially, but ongoing reassessment is crucial once additional data (e.g., imaging, serologies) become available.
5. Temporary pacing may be sufficient in cases of myocarditis‐related heart block, with many patients recovering normal conduction as myocardial inflammation resolves.

## 1. Background

Myocarditis is a rare complication after mRNA COVID‐19 vaccination, mostly in young males. Symptoms of myocarditis due to cardiac‐specific cellular and humoral inflammation commonly occur within several days of vaccination. However, cases with later onset ranging up to 3 weeks have also been reported [1]. Cases in older adults are less common and often atypical [1]. The proposed pathophysiology involves immune‐mediated myocardial inflammation triggered by the vaccine, resulting in clinical manifestations that may mimic acute coronary syndrome (ACS), including chest discomfort, elevated cardiac biomarkers, and electrocardiogram (ECG) changes, despite nonobstructive coronary arteries [1, 2].

Though often mild and self‐limited, myocarditis can sometimes cause conduction issues like high‐grade atrioventricular (AV) block or more serious issues like ventricular arrhythmias or heart failure [1, 3]. Diagnosis requires a high index of suspicion and often uses cardiac magnetic resonance imaging (cMRI), the noninvasive gold standard for tissue analysis. However, it is not routinely done, especially in older patients or those with atypical symptoms [1, 3].

We report an elderly woman with a complete heart block diagnosed with myocarditis following a recent Moderna COVID‐19 booster.

## 2. Case Report

### 2.1. History of Presentation

A woman in her late 70s came to the emergency department with a 3‐day history of worsening exertional dyspnea and intermittent substernal chest pressure. The symptoms started while carrying groceries and were initially relieved by rest, but later worsened to shortness of breath with minimal activity. She denied orthopnea, paroxysmal nocturnal dyspnea, syncope, fever, or gastrointestinal symptoms. She reported a dry cough and nasal congestion but tested negative for COVID‐19 on two home tests. Concerned, she visited urgent care, where an ECG showed a complete heart block. She was then transferred to the emergency department by emergency medical services.

### 2.2. Past Medical History

Her medical history includes hypertension, managed with a stable antihypertensive regimen. She has no diabetes or cardiac conditions, including coronary disease. Her family history is unremarkable. She is married, self‐employed, active, abstains from tobacco, alcohol, and drugs, and has no known allergies.

### 2.3. Investigations

**Figure 1:**
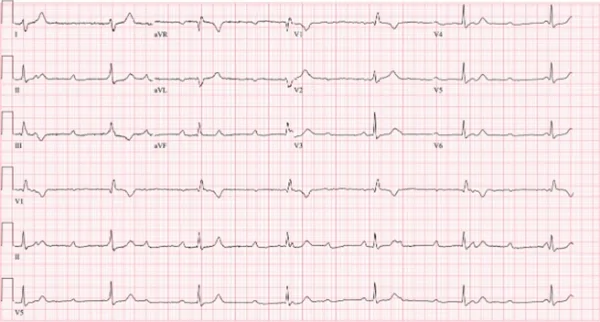
Electrocardiogram at presentation with sinus rhythm and third‐degree atrioventricular block.

On arrival, she was alert and oriented;hemodynamically stable with a heart rate in the 50s. ECG confirmed third‐degree AV block (Figure 1). Laboratory workup showed elevated high‐sensitivity troponin I of 16.8 ng/L, peaking at 1478.5 ng/L (ref range ≤ 15.0 pg/mL), B‐type natriuretic peptide of 384 pg/mL, and mildly elevated transaminases (aspartate aminotransferase 86 U/L and alanine aminotransferase 191 U/L). Electrolytes and renal function were normal. Chest x‐ray was unremarkable. A temporary transvenous pacemaker (TVP) was placed via the femoral vein, and emergent left heart catheterization revealed normal coronary arteries without obstructive disease. Echocardiogram showed left ventricular ejection fraction (LVEF) 40%–45%, regional wall motion abnormalities in the anteroseptum and apex, and Grade I diastolic dysfunction.

### 2.4. Differential Diagnosis

Given the lack of coronary artery disease and elevated troponin, other differentials were considered, especially Lyme carditis initially, given the high prevalence of tick‐borne illnesses in the region and the patient′s presentation. Stress‐induced (Takotsubo) cardiomyopathy was considered given her demographics, myocarditis, and cardiac sarcoidosis given the new high‐grade AV block and nonischemic myocardial enhancement on cardiac MRI. Infectious disease (ID) and electrophysiology (EP) services were consulted early in the hospitalization. The ID team recommended empiric treatment with intravenous ceftriaxone for possible Lyme carditis while serologic testing was pending. EP confirmed complete heart block with a narrow escape rhythm and recommended ongoing observation with temporary pacing support, as there was a high suspicion of Lyme carditis due to its prevalence and anticipated improvement with treatment. Over the next 2 days, the patient′s rhythm gradually improved from complete heart block to intermittent second‐degree AV block, eventually transitioning to a stable sinus rhythm. Further infectious and inflammatory workup, including Lyme serologies, tick‐borne panels, *Bartonella* polymerase chain reaction (PCR) and serology (due to a recent cat bite), and chest computed tomography (CT), was unrevealing. The patient also denied any history of tick exposure or travel to endemic areas.

**Figure 2:**
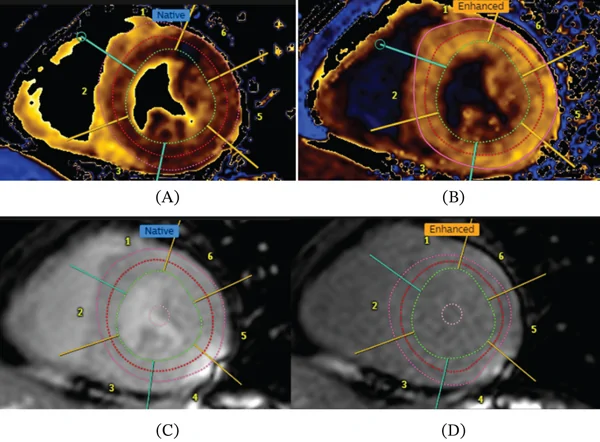
Cardiac MRI showing (A) native (noncontrast) T1 mapping, (B) enhanced (postcontrast) T1 mapping, (C) native (noncontrast) extracellular volume (ECV) mapping, and (D) enhanced (postcontrast) ECV mapping.

**Figure 3:**
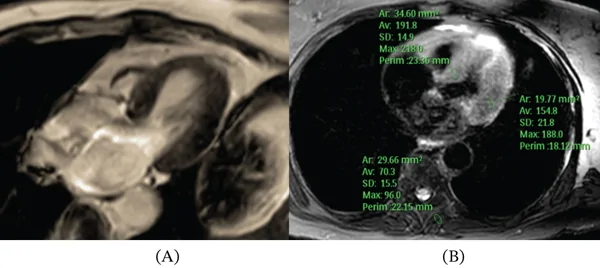
Cardiac MRI showing (A) aortic outflow long axis plane demonstrating patchy, mid‐myocardial delayed enhancement predominantly in the mid to distal septum and anterior wall and (B) edema sequence comparing myocardium signal intensity with skeletal muscle.

The cMRI showed acute myocarditis per 2018 Lake Louise Criteria, with elevated native T1 (average 1358 ms, Zone 1 1419 ms, and Zone 2 1414 ms) (Figure 2A,B), patchy nodular mid‐myocardial late gadolinium enhancement (Figure 3A), and increased T2 values (average 36%; 36% in Zone 1 and 38% in Zone 2) (Figure 2C,D), indicating edema. The edema sequence ratio was 2.27 (218/96) (Figure 3B). Abnormal findings included elevated T1/T2, ECV, septal enhancement, and regional wall motion abnormalities with a reduced LVEF of 45%, supporting inflammatory myocarditis rather than ischemia. Cardiac sarcoidosis was less likely without pulmonary or lymphatic signs.

### 2.5. Treatment

**Figure 4:**
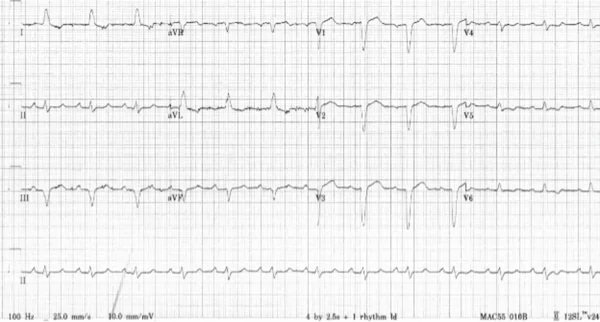
EKG 4 days later showing stable sinus rhythm and resolution of complete atrioventricular block with left axis deviation and LBBB.

The patient continued improving and remained free of high‐grade AV block (Figure 4). The TVP was removed after 2 days, and placement of a permanent pacemaker was deferred given the progressive recovery of AV conduction, hemodynamic stability, and absence of recurrent high‐grade AV block. A detailed vaccination history revealed that she had received six COVID‐19 vaccine doses, including a three‐dose Pfizer‐BioNTech series followed by three Moderna vaccinations, one of which was a bivalent formulation. She had tolerated all previous doses without adverse reactions. Her most recent dose was a Moderna COVID‐19 booster (0.5 mL) 6 days before symptom onset. Given this temporal relationship, together with an unrevealing infectious and autoimmune evaluation and normal coronary anatomy, a possible association between the recent vaccination and acute myocarditis was considered. She did not develop ventricular arrhythmias or cardiogenic shock; steroids or immunosuppressants were not given as symptoms resolved with supportive care. Guideline‐directed medical therapy (GDMT) was held; she was advised to avoid strenuous activity for 3–6 months. Follow‐up plans include repeat cardiac function assessment and GDMT titration as an outpatient.

### 2.6. Outcome and Follow‐up

**Figure 5:**
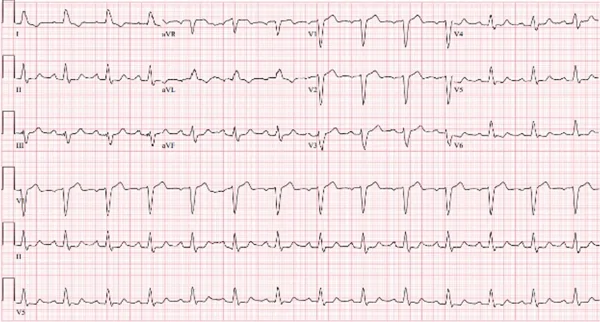
EKG at 1‐month follow‐up after hospital discharge showing a stable sinus rhythm with left axis deviation and LBBB.

The conduction abnormality was considered potentially reversible in the setting of acute myocarditis, and she was discharged in stable condition with follow‐up through outpatient EP and a 30‐day wearable cardiac monitor. Cardiac positron emission tomography imaging was considered if concern for sarcoidosis persisted. At her follow‐up, she felt well and had returned to baseline. Her ECG showed normal sinus rhythm with a left bundle branch block, and she remained stable with monitoring (Figure 5). No concerning arrhythmias were found, and no additional tests were needed after the monitor. She completed a year of follow‐up and is doing well. At 4 months, a gated myocardial ventriculogram showed improved LVEF (63%) with no regional wall hypokinesis. A detailed timeline of the patient is provided in Table 1.

**Table 1:** Clinical timeline of complete atrioventricular block and recovery following COVID‐19 vaccination.

Clinical course	Rhythm/clinical findings	Key investigations/management
6 days before symptom onset	Received most recent Moderna COVID‐19 booster	Previous COVID‐19 vaccinations had been well tolerated without adverse reactions.
Symptom onset	Exertional dyspnea and intermittent substernal chest pressure	Symptoms progressed over 3 days
Presentation	Complete (third‐degree) AV block with narrow escape rhythm	Temporary transvenous pacemaker placed; coronary angiography showed no obstructive CAD
Following 2 days	Complete heart block → intermittent second‐degree AV block → sinus rhythm	Temporary pacing and close rhythm monitoring; empiric ceftriaxone while Lyme testing was pending
During hospitalization	Progressive recovery of AV conduction	Temporary pacemaker removed; cardiac MRI fulfilled criteria for acute myocarditis; infectious and inflammatory workup unrevealing.
Before discharge	Stable sinus rhythm without recurrent high‐grade AV block	Permanent pacemaker deferred
Discharge	Stable rhythm	30‐day ambulatory cardiac monitor and EP follow‐up
1‐month follow‐up	Sinus rhythm with LBBB; no concerning arrhythmias on monitoring	Continued outpatient observation
4‐month follow‐up	Clinically well; LVEF improved to 63%	Continued follow‐up
1‐year follow‐up	Clinically well	No further intervention required

## 3. Discussion

Myocarditis after mRNA COVID‐19 vaccination is a rare but important side effect, mainly affecting young males with chest pain, elevated cardiac biomarkers, and normal ventricular function [1, 2]. As vaccine uptake increased across all ages, reports show a broader clinical spectrum of postvaccination myocarditis, including arrhythmias and conduction disturbances [1, 4, 5]. Although uncommon, high‐grade AV block has been reported in the context of vaccine‐associated myocardial inflammation and poses unique diagnostic and management challenges [4, 5].

Here, a woman in her late 70s presented with exertional dyspnea and complete heart block, initially raising concern for ACS or Lyme carditis. Coronary angiography showed no evidence of obstruction, and the infectious and inflammatory workup, including Lyme serologies and *Bartonella* testing, was negative. The cMRI supported a myocarditis diagnosis with mid‐myocardial delayed enhancement indicating inflammation. Symptoms began 6 days after a COVID‐19 booster vaccination, raising suspicion of vaccine‐related etiology given the lack of other causes.

Vaccine‐associated myocarditis likely results from an immune response targeting heart tissue, triggered by molecular mimicry or activation of innate immunity after exposure to vaccine mRNA or lipid nanoparticles [1, 6]. Histologic findings in reported cases have included lymphocytic infiltrates consistent with acute myocarditis [2, 6]. The association between symptom onset and vaccination, usually about a week after, suggests a causal link in some cases. Most cases are mild and self‐limited, but severe presentations like cardiogenic shock and complete heart block have been reported [4, 5].

Two recent reports describe transient complete heart block in healthy adults after COVID‐19 vaccination, resolving spontaneously as clinical and myocardial inflammation improve [7, 8], without the need for any anti‐inflammatory medications or steroid therapy. Our patient has a distinct phenotype: elderly (> 75 years), initial complete heart block, myocarditis confirmed by cMRI, and spontaneous recovery of AV conduction without permanent pacing. Although earlier reports involve younger patients, fulminant myocarditis, recurrent conduction issues requiring pacemakers, or resolution after immunosuppression, fully reversible complete AV block in an elderly patient without pacing is very rare (Table 2). This case broadens the spectrum of vaccine‐associated myocardial inflammation, showing rhythm normalization without permanent pacing. It highlights the importance of conservative management once reversible causes are excluded and myocarditis is suspected, and ultimately avoiding unnecessary permanent pacing.

**Table 2:** Case reports reported thus far reporting COVID‐19 vaccine‐associated myocarditis and heart block.

Author (year)	Age/sex	Vaccine type	Presentation	Conduction abnormality	Imaging confirmation	Outcome
Etienne et al., 2022 [5]	73/M	mRNA	Syncope, dizziness	Recurrent complete AV block	Not clearly CMR‐confirmed	Pacemaker implanted despite partial recovery
Kimball et al., 2022 [7]	57/M	Pfizer	Syncope	Intermittent complete AV block with ventricular standstill	CMR consistent with myocarditis	Temporary pacing → recovery
Pons‐Riverola et al., 2022 [8]	49/M	Pfizer	Bradycardua	Complete AV block	Suspected Myocarditis	Improved with steroids
Onishi et al., 2023 [4]	71/M	Pfizer	Cardiogenic shock	Complete AV block	CMR + biopsy	Fulminant course

The cMRI was crucial for establishing the diagnosis, as electrocardiography, cardiac biomarkers, and echocardiography lacked specificity. Despite its diagnostic value, cardiac MRI remains underused in suspected myocarditis, particularly in older adults with atypical presentations [1]. A high index of suspicion should be maintained in patients presenting with new high‐grade conduction abnormalities. Early recognition of myocarditis is particularly important because associated conduction disturbances may be reversible as myocardial inflammation resolves, allowing recovery of AV conduction and potentially avoiding unnecessary permanent pacemaker implantation.

Although vaccine‐associated myocarditis raises concern, it should be viewed in the context of the overall benefits and risks of COVID‐19 vaccination. The incidence remains low, and most cases resolve without long‐term sequelae [1, 2]. Continued surveillance and clinician awareness are vital for the timely recognition and management of these rare complications and potentially avoid unnecessary permanent pacemaker implantation.

**Nomenclature**
ACS: acute coronary syndrome
AV: atrioventricular
cMRI: cardiac magnetic resonance imaging
COVID‐19: coronavirus disease‐2019
ECV: extracellular volume
GDMT: guideline‐directed medical therapy
TVP: temporary transvenous pacemaker

## Funding

No funding was received for this manuscript.

## Disclosure

This study is not commissioned and is externally peer reviewed.

## Ethics Statement

This case report was prepared with approval from the institutional department. In accordance with institutional policy, ethical review was not required for a single‐case report with patient consent.

## Consent

Written informed consent has been obtained from the patient for publication of this case report and any accompanying images.

## Conflicts of Interest

The authors declare no conflicts of interest.

## Data Availability

The data that support the findings of this study are available on request from the corresponding author. The data are not publicly available due to privacy or ethical restrictions.
